# PM00104 (Zalypsis^®^): A Marine Derived Alkylating Agent

**DOI:** 10.3390/molecules190812328

**Published:** 2014-08-15

**Authors:** Bradley J. Petek, Robin L. Jones

**Affiliations:** 1 University of Washington School of Medicine, 1959 NE Pacific St, Seattle, WA 98195, USA; E-Mail: bpetek@uw.edu; 2 Fred Hutchinson Cancer Research Center, University of Washington, 825 Eastlake Avenue East, G-3630, Seattle, WA 98109-1023, USA

**Keywords:** PM00104, Zalypsis, alkaloid, chemotherapy, cancer therapy

## Abstract

PM00104 (Zalypsis^®^) is a synthethic tetrahydroisoquinolone alkaloid, which is structurally similar to many marine organisms. The compound has been proposed as a potential chemotherapeutic agent in the treatment of solid human tumors and hematological malignancies. PM00104 is a DNA binding agent, causing inhibition of the cell cycle and transcription, which can lead to double stranded DNA breaks. After rigorous pre-clinical testing, the drug has been evaluated in a number of phase II clinical trials. This manuscript provides a review of current trials and appraises the efficacy of PM00104 as a future cancer treatment.

## 1. Introduction

Natural compounds have been very important in the development of medicine throughout history. Advancements in science have allowed researchers to screen millions of naturally occurring substances in the environment for the potential of creating effective new therapies. Many successful drugs have been developed through mimicry of toxins found in plants and marine organisms. One field that has been directly impacted through wide-scale screens of natural compounds is oncology. Harnessing the potential utility of these substances may prove to be a fruitful approach in cancer therapy.

Alkaloids are a large group of cyclic compounds among these naturally occurring substances that have been especially important in modern cancer therapy. Some common alkaloids that have been used as effective chemotherapy treatments include: Vincristine, Paclitaxel, Docetaxel, Etoposide, and Irinotecan. These chemicals typically contain a basic nitrogen from an amino acid, and have the ability to inhibit cell proliferation at many different levels [1].

PM00104 (Zalypsis^®^) is a synthetic tetrahydroisoquinolone alkaloid, which mimics many natural marine compounds. The drug is structurally similar to jorumycin (isolated from a Pacific nudibranch), ecteinascidins (isolated from tunicates), renieramycins (isolated from sponges), safracins and saframycins (isolated from bacteria and sponges) [2,3,4,5]. PM00104 has shown strong initial *in vitro* models as a chemotherapeutic agent, and has had positive outcomes within *in vivo* experiments on a wide variety of human tumors. More recently, PM00104 has progressed into phase II clinical trials to treat Ewing sarcoma, urothelial carcinoma, multiple myeloma, endometrial and cervical cancer.

## 2. Mechanism of Action

PM00104 utilizes its reactive carbinolamine group to bind to the minor groove of DNA. The drug covalently bonds to guanine residues with a preferential affinity for AGG, GGC, AGC, CGG, and TGG groups [6]. The molecular interaction between PM00104 and DNA leads to the creation of a DNA adduct which inhibits the early phases of transcription, and also causes double-stranded DNA breaks to occur [6,7]. PM00104 affects the cell cycle by inducing S-phase arrest and eventually apoptosis. The versatility of PM00104 to inhibit cell proliferation by many different modalities has led to an increased interest in the possible clinical utility of the drug.

With the potential for use of PM00104 in clinical treatment, identification of novel biomarkers for the effectiveness of the drug has also become important. The activation of tyrosine kinase receptors (RTKs) has been proposed to determine the susceptibility of tumors to PM00104. All cell lines with low levels of tyrosine kinase receptor signaling were sensitive to PM00104 *in vivo*, and higher levels of RTK activation conferred resistance [8]. Another study has also suggested the gamma-H2AX foci as a potential pharmacodynamic biomarker for PM00104 [9]. Since there is limited data on biomarkers for this drug, future research in this field will be important in the development of PM00104.

**Figure 1:**
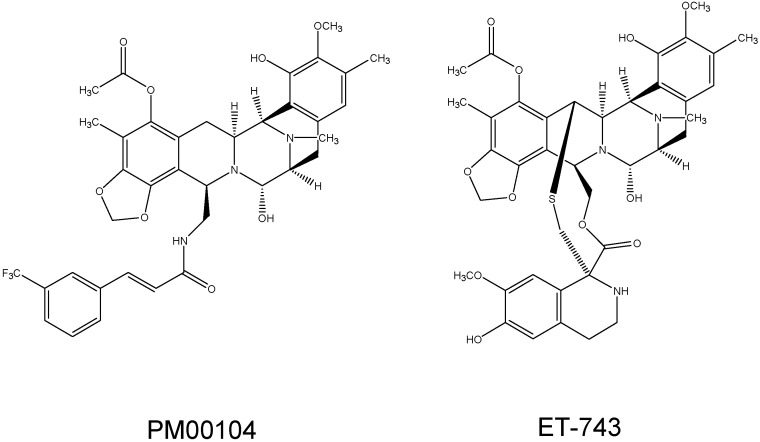
Chemical structures of PM00104 and ET-743.

Active research on another chemotherapeutic agent known as ET-743 (trabectedin), has led to a greater understanding of the mechanistic capabilities of PM00104. Trabectedin is a natural extract from the tunicate *Ecteinascidia turbinata*, and another alkaloid used in cancer treatment [5]. ET-743 has the same cyclic structure of PM00104, although it differs a little in one of its rings (Figure 1). This alteration in structure causes the two drugs to have slightly different DNA binding properties and nucleotide excision repair dependencies [9]. Otherwise, the mechanisms of their cytotoxic action are similar. Each drug has been effective in reducing cell proliferation in multiple cancer cell lines.

## 3. Preclinical Development

PM00104 has shown significant *in vitro* activity against both solid and non-solid human tumors. The chemotherapeutic agent has exhibited antitumor activity to cell lines of bladder, gastric, kidney, prostate, pancreatic, and thyroid cancers, as well as in melonoma, sarcoma, leukemia, and lymphoma [6,10,11]. Half-maximal inhibitory concentrations (IC_50_) were calculated to demonstrate activity against these various cell types. Successful *in vitro* studies propelled research into *in vivo* murine models. PM00104 has been able to treat breast, liver, prostate, bladder, gastric, and pancreatic tumors in xenografted mice [12,13,14,15]. The success in preclinical development of PM00104 led to subsequent clinical trials.

## 4. Phase I Trials

Initial phase I clinical trials for PM00104 have primarily been set up as dose escalation studies to assess the toxicity of the drug. Pharmacokinetic analysis of these preliminary experiments has established effective dosing schedules for intravenous administration [16,17,18]. The current recommended treatment of PM00104 in phase II trials is 2 mg/m^2^ as a 1 h infusion on days 1, 8, and 15 of a 4-week cycle [17].

All of the phase I clinical trials to date have reported adverse events as only mild or moderate while under appropriate dosing of PM00104. Common side effects from the chemotherapy treatment have included nausea, fatigue, and vomiting [16,17,18]. Many patients also present with significant biochemical and laboratory abnormalities after infusions. The most prevalent findings include myelosuppression and elevations in transaminases. With administration of PM00104, the noted adverse events and laboratory tests should be carefully monitored.

Model based simulations comparing phase I clinical trials have found that PM00104-induced neutropenia is mainly dependent on the dosing frequency and concentration of the drug [19]. The duration of intravenous infusions appeared to have no effect on patients. Neutropenia can therefore be potentially bypassed by employing treatment regimens with more frequent dosing, while the overall amount of the drug delivered remains the same [19].

While almost all of these studies have used PM00104 as a single-agent therapy, one recent trial used the drug in combination with carboplatin [20]. The results of the study confirmed that combination therapy with PM00104 has the potential for overlapping toxicities, which hampers the dose escalation of either compound. Although multiple drugs may have to be administered at lower concentrations, the benefit of combination therapies has been widely accepted in the treatment of many types of cancer. With success in preliminary studies, PM00104 has moved into phase II clinical trials.

## 5. Phase II Trials

### 5.1. Ewing Sarcoma

Ewing sarcoma is a rare bone and soft tissue cancer that most often affects children and adolescents. The disease is characterized by rearrangements between the EWS gene and members of the ETS gene family [21].

The main goal of this study was to measure the objective response rates of patients with Ewing sarcoma to PM00104 [22]. The treatment schedule of the drug was 2 mg/m^2^ as a 1 h infusion on days 1, 8, and 15 of a 4-week cycle. The study concluded that there were no objective responses in the cohort of 16 evaluable patients with Ewing sarcoma. The median progression-free survival rate was recorded as 1.8 months. No further trials with PM00104 at this dosing schedule will be performed in this population since the study showed no clear benefit of using the drug. Active research for well-tolerated, novel therapies will be needed to appropriately treat future patients presenting with advanced/metastatic forms of Ewing sarcoma.

### 5.2. Urothelial Carcinoma

Urothelial (transitional cell) carcinoma is a type of cancer directly affecting the urinary tract. Bladder cancer is the ninth most prevalent malignancy worldwide, and urothelial carcinoma is the most common subtype found in the United States and Western Europe [23]. Cigarette smoking and occupational carcinogens are the biggest risk factors for developing the disease [23,24].

This phase II clinical trial was run on patients with urothelial carcinoma who were progressing after first-line platinum-based chemotherapy [25]. PM00104 was administered at 3 mg/m^2^ for 1 h every 3 weeks. In the first stage of the study, only 1 of 19 patients achieved an objective response or progression-free cancer at 3 months. The study was terminated early because there seemed to be no benefit from the current therapy.

### 5.3. Endometrial and Cervical Cancer

Endometrial and cervical cancers are the first and third most common gynecological malignancies affecting women [26]. Prevalent risk factors for cervical cancer include early onset of sexual activity, and having multiple sexual partners [27]. Some of the main risk factors for endometrial cancer include long-term estrogen therapy, late menopause, and obesity [28].

This clinical trial was an open-label, two-arm study designed to assess the antitumor activity and safety concerns with the use of PM00104 in advanced/metastatic endometrial or cervical cancer [29]. All of the patients included in the study were also required to have had one previous administration of chemotherapy. The treatment schedule of PM00104 was 2 mg/m^2^ as a 1 h infusion on days 1, 8, and 15 of a 4-week cycle. After two cycles of treatment for 12 patients with endometrial cancer (EC), and 7 patients with cervical cancer (CC), none achieved an objective tumor response. Median progression-free survival was 1.8 months for EC patients and 1.5 for CC patients. The median overall survival rate was 5.5 months for EC patients and 5.6 months for CC patients. Overall, PM00104 as a single-agent did not prove to be an effective therapy for patients with advanced/metastatic endometrial or cervical cancer.

### 5.4. Multiple Myeloma

Multiple myeloma is a hematological form of cancer caused by the neoplastic proliferation of plasma cells producing a monoclonal immunoglobulin. Patients often present with bone pain and lytic lesions on skeletal films, but many other systemic symptoms can present. Multiple myeloma accounts for slightly more than 10% of hematologic cancers in the United States [26].

This study was conducted to determine the recommended dose of PM00104 to be administered as a 1 h infusion on days 1, 8, and 15 every 4 weeks in relapsed or refractory multiple myeloma patients [30]. The complete results from this trial have not been published, but preliminary data showed PM00104 to be safe to use in the treatment of multiple myeloma. The recommended dose of PM00104 for this regimen was also established at 2 mg/m^2^. This same treatment scheme has been used in other phase II clinical trials, and has also been evaluated in phase I trials [17,22,29].

## 6. Conclusions and Future Perspectives

While PM00104 has shown promising single agent anti-tumor activity in laboratory studies, this has not translated into clinical benefit in a number of phase II trials. Further studies should consider combination schedules of the drug. The phase I trial of PM00104 and carboplatin demonstrated overlapping toxicity, and consequently no phase II trials with combination therapy have been performed. PM00104 has the potential to be beneficial when paired with other chemotherapeutic agents, and the possibility of combination schedules with immunotherapy is another interesting approach for further exploration. Upcoming trials should also incorporate imaging or molecular markers of response, in order to delineate patients most likely to benefit from this drug. There is a clear unmet need for novel effective schedules in diseases such as Ewing sarcoma. Therefore, future trials should focus on these tumor types where limited effective systemic therapies currently exist. The potential utility of this drug in combination schedules remains unanswered, and should be considered for future research.
